# Genal: a Python toolkit for genetic risk scoring and Mendelian randomization

**DOI:** 10.1093/bioadv/vbae207

**Published:** 2024-12-24

**Authors:** Cyprien A Rivier, Santiago Clocchiatti-Tuozzo, Shufan Huo, Victor Torres-Lopez, Daniela Renedo, Kevin N Sheth, Guido J Falcone, Julian N Acosta

**Affiliations:** Department of Neurology, Yale School of Medicine, New Haven, CT 06510, United States; Yale Center for Brain and Mind Health, Yale School of Medicine, New Haven, CT 06510, United States; Department of Neurology, Yale School of Medicine, New Haven, CT 06510, United States; Yale Center for Brain and Mind Health, Yale School of Medicine, New Haven, CT 06510, United States; Department of Neurology, Yale School of Medicine, New Haven, CT 06510, United States; Yale Center for Brain and Mind Health, Yale School of Medicine, New Haven, CT 06510, United States; Department of Neurology, Yale School of Medicine, New Haven, CT 06510, United States; Department of Neurology, Yale School of Medicine, New Haven, CT 06510, United States; Yale Center for Brain and Mind Health, Yale School of Medicine, New Haven, CT 06510, United States; Department of Neurology, Yale School of Medicine, New Haven, CT 06510, United States; Yale Center for Brain and Mind Health, Yale School of Medicine, New Haven, CT 06510, United States; Department of Neurology, Yale School of Medicine, New Haven, CT 06510, United States; Yale Center for Brain and Mind Health, Yale School of Medicine, New Haven, CT 06510, United States; Department of Biomedical Informatics, Harvard Medical School, Boston, MA 02115, United States

## Abstract

**Motivation:**

The expansion of genetic association data from genome-wide association studies has increased the importance of methodologies like Polygenic Risk Scores (PRS) and Mendelian Randomization (MR) in genetic epidemiology. However, their application is often impeded by complex, multi-step workflows requiring specialized expertise and the use of disparate tools with varying data formatting requirements. Existing solutions are frequently standalone packages or command-line based—largely due to dependencies on tools like PLINK—limiting accessibility for researchers without computational experience. Given Python’s popularity and ease of use, there is a need for an integrated, user-friendly Python toolkit to streamline PRS and MR analyses.

**Results:**

We introduce Genal, a Python package that consolidates SNP-level data handling, cleaning, clumping, PRS computation, and MR analyses into a single, cohesive toolkit. By eliminating the need for multiple R packages and for command-line interaction by wrapping around PLINK, Genal lowers the barrier for medical scientists to perform complex genetic epidemiology studies. Genal draws on concepts from several well-established tools, ensuring that users have access to rigorous statistical techniques in the intuitive Python environment. Additionally, Genal leverages parallel processing for MR methods, including MR-PRESSO, significantly reducing the computational time required for these analyses.

**Availability and implementation:**

The package is available on Pypi (https://pypi.org/project/genal-python/), the code is openly available on Github with a tutorial: https://github.com/CypRiv/genal, and the documentation can be found on readthedocs: https://genal.rtfd.io.

## 1 Introduction

As the volume of genetic association data from genome-wide association studies (GWAS) continues to expand, methodologies such as Polygenic Risk Scores (PRS) and Mendelian Randomization (MR) analyses have rapidly gained importance within and beyond the field of genetic epidemiology. PRS aggregates the effects of numerous genetic variants across the genome, each contributing a small effect size, to create a comprehensive score that quantifies an individual’s genetic predisposition to various diseases and traits. By using GWAS summary statistics to weigh these variants based on their association strengths, PRS can effectively stratify individuals based on their genetic risk, facilitating advancements in risk prediction, preventive strategies, and personalized medicine (Lewis and Vassos 2020).

Concurrently, MR employs genetic variants as instrumental variables to establish causal relationships between traits (exposures) and clinical outcomes. By assuming that these genetic instruments are associated with the exposure of interest but not directly with the outcome except through the exposure, MR can mitigate confounding and reverse causation inherent in traditional observational studies. This robust approach enables researchers to identify potential therapeutic targets, understand disease mechanisms, and prioritize interventions with greater confidence, thereby enhancing the translational impact of genetic research (Richmond and Davey Smith 2022).

Despite the increasing utility of PRS and MR in leveraging genetic association data for clinical and research purposes, their application is often hindered by the multi-step processes involved in data preparation and analysis. The need for specialized knowledge in bioinformatics and the use of disparate tools for different steps of the analytical process, each requiring preparing the data in a different way, further complicate the workflow, making it less accessible to researchers with non-computational backgrounds.

Python is currently the most popular programming language according to the TIOBE Programming Community index [December 2024 ranking (“TIOBE Index, 2024”)]. Its ascent to prominence is largely attributable to its simplicity, readability, and adaptability, which allows for the seamless integration of statistical analyses, machine learning algorithms, and data visualization. In this context, we present Genal, a Python toolkit that integrates Single-Nucleotide Polymorphisms (SNP)-level data handling, cleaning, clumping, lifting, SNP association testing, Polygenic Risk Scoring, and MR functionalities into one user-friendly package. While some Python-based tools offer to run PRS through a friendly interface (Page *et al.* 2022), or can run MR (Dalal 2024), they often exist as standalone scripts dedicated to one particular methodology and do not integrate well with other workflows. Furthermore, MR analyses are currently predominantly conducted using R packages like TwoSampleMR (Hemani *et al.* 2018), which can be a barrier for many users who have been introduced to programming through Python-based environments.

Genal distinguishes itself by consolidating these functionalities into a single, cohesive Python package. By integrating SNP-level data cleaning, clumping, PRS, MR, and other convenient functionalities within one user-friendly package, Genal streamlines the analytical workflow for researchers and clinicians. This integration reduces the need to switch between multiple packages with various dependencies, minimizing compatibility issues and learning curves associated with disparate software. It also entirely avoids the need to interact with the command line, making advanced genetic analyses more accessible to users with varying levels of programming expertise, thereby enhancing efficiency and promoting reproducibility in genomic research.

## 2 Implementation

### 2.1 The *Geno* class

**Figure 1.**
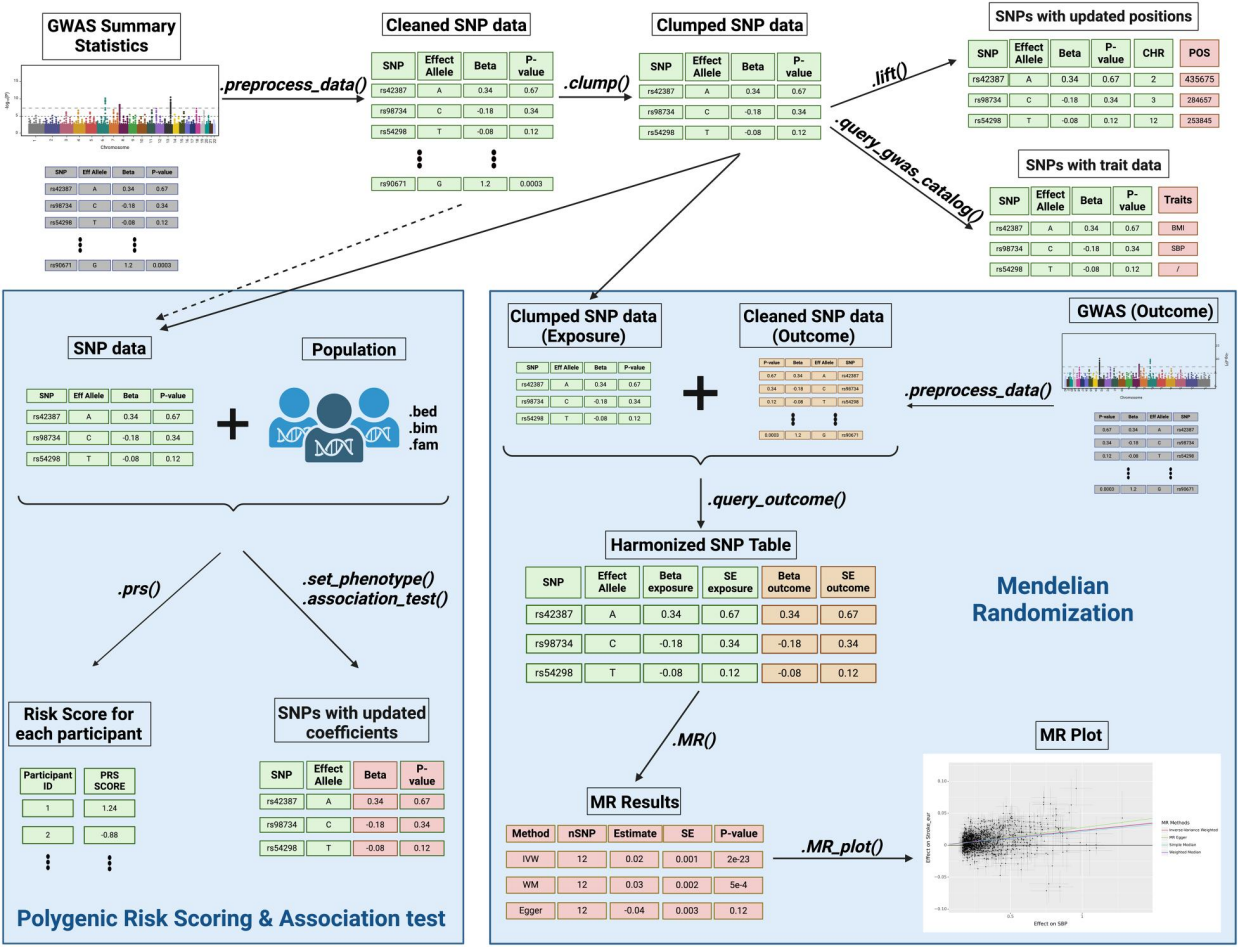
Flowchart describing the main functionalities of Genal. Created in BioRender. Falcone, G. (2024) https://BioRender.com/q72r861.

The ‘*Geno*’ class is the central element of the Genal package (Fig. 1). It is designed to store SNP association data, and all functionalities of the toolkit are implemented as methods of this class. The main attribute of the ‘*Geno*’ class is a pandas DataFrame (The pandas development team 2024), which stores the information for each SNP as rows and includes columns for the genomic position, reference SNP cluster ID (rsid), point estimate, standard error, and *P*-value of the association with the trait, effect and non-effect alleles, and the frequency of the effect allele. Not all columns must be present to create an instance of the ‘*Geno*’ class. If some of them are missing, the ‘*preprocess_data*’ method can derive it from the existing columns and genetic reference panels. For example, it can determine the genomic position from the rsid, or conversely, ascertain the rsid from the genomic position. Similarly, the standard error can be deduced from the *P*-value and vice versa. The ‘*preprocess_data*’ method also performs a variety of checks to clean the data, eliminating errors, missing values, or invalid entries across each column. It ensures the data are formatted correctly for use in the toolkit’s downstream methods. Users have the flexibility to customize this list of checks. The method provides feedback by displaying stepwise results, allowing users to monitor the number of SNPs filtered out at each stage.

### 2.2 Clumping and risk scoring

After SNP data are loaded into a ‘*Geno*’ instance and have been cleaned and formatted, several genetic methods can be applied directly with just a single line of code. The clump method allows for clumping, returning a new Geno instance with data clumped based on specified thresholds and reference panels. To calculate a PRS for a population, the ‘*prs*’ method is used, providing the option to use proxies at specified thresholds. If the genetic data is divided into separate files for each chromosome, Genal manages the extraction and merging of the required SNPs. The PRS method can be applied to any Geno instance, whether it has been processed through clumping or not. Both the clumping and PRS methods are implemented as wrappers around Plink (Purcell *et al.* 2007), and Genal can automatically install the appropriate version. By default, the reference panels used are from the 1000 Genomes Project, but users can provide paths to their own custom panels if desired.

### 2.3 Mendelian randomization

**Figure 2.**
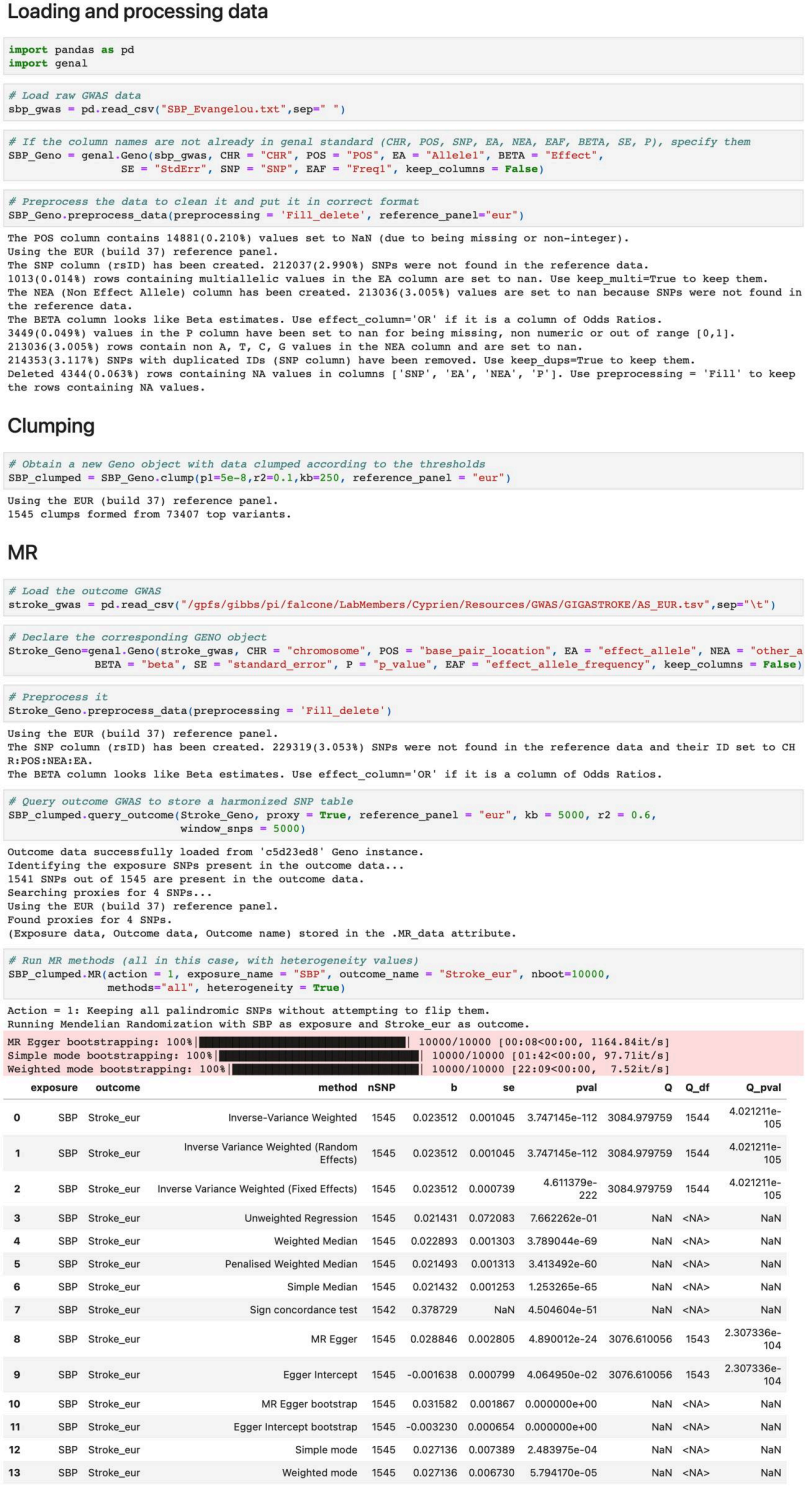
Screenshot of a Jupyter notebook showing the steps to run MR.

**Figure 3.**
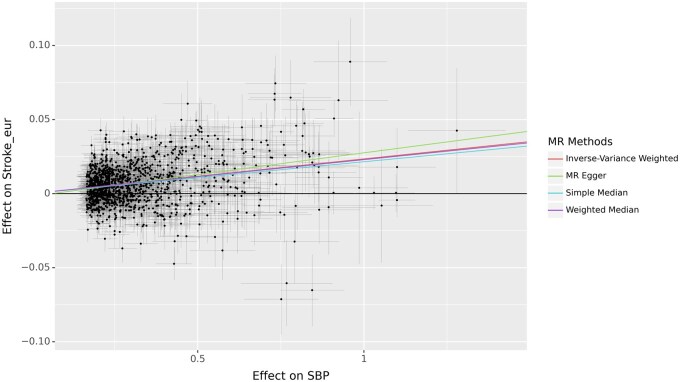
Figure produced by the *MR_plot* function to visualize the results of Mendelian Randomization analyses.

Running MR with Genal is straightforward, and begins with the preparation of two ‘*Geno*’ instances (Fig. 2). The first should include SNPs selected as instruments along with their association values for the exposure trait. This is typically a ‘*Geno*’ instance resulting from the clumping step. The second ‘*Geno*’ instance should contain summary statistics for the association with the outcome trait, which is used to extract the values corresponding to the instruments. After formatting both Geno instances with ‘*preprocess_data*’, the necessary dataframe for MR is generated by invoking the ‘*query_outcome*’ method on the ‘*Geno*’ instance containing the exposure data. The combined dataframe is stored as an attribute. In cases where not all instruments are present in the outcome data, the proxy option allows for the use of alternate SNPs within specified thresholds. To conduct MR analysis, one simply calls the ‘*MR*’ method. This method takes care of SNP harmonization and deals with palindromic SNPs (the nucleotide sequence is identical when read forward and backward) as per the user’s specifications, similar to the process outlined in TwoSampleMR (Hemani *et al.* 2017). Users can specify which MR methods to run, along with advanced settings such as the number of bootstrapping iterations for particular MR analyses, or options to return heterogeneity results. The outcomes of the MR analysis are presented in a table format and can be visually represented using the ‘*MR_plot*’ method (Fig. 3). To address potential horizontal pleiotropy, in addition to the weighted median and MR-Egger methods available within the ‘*MR*’ method, the MR-PRESSO (Verbanck *et al.* 2018) algorithm can be invoked via the ‘*MRpresso*’ method. All the functionalities mentioned are fully implemented in Python, and the bootstrapping and MR-PRESSO methods utilize parallel processing to speed up the execution.

### 2.4 Customization

Though Genal was designed to be user-friendly, we also emphasized flexibility, allowing users to customize most functions to cater to a wide range of genetic studies across different species. Parameters and thresholds, such as *P*-value cut-offs, linkage disequilibrium thresholds, and genomic window sizes, default to widely accepted values in human genetics. However, users can adjust these settings to better align with their specific study designs. Similarly, all parameters of the different MR functions can be customized, including the number of bootstrapping iterations or the bandwidth parameter in mode methods. Additionally, users can provide their own custom reference panels in PLINK format to accommodate non-human genetics or human populations not represented in standard reference datasets like the 1000 Genomes Project.

Despite our efforts to make the package as user-friendly as possible, we acknowledge that obtaining high-quality genetic datasets, custom linkage disequilibrium panels, or PRS weights and effect sizes may still involve command-line interactions, especially if performed on computing clusters rather than personal computers. To mitigate this, Genal provides detailed documentation and tutorials to assist users in integrating their data into the workflow.

### 2.5 Future directions

Moving forward, we aim to optimize our existing functionalities and introduce new features to enhance both performance and user experience. At present, the primary bottleneck in computing efficiency lies in managing large datasets, particularly the reference panels. Loading data into memory and executing merge operations on the 1K genome panels are the most time- and memory-consuming aspects of our data processing pipeline. To accommodate newer panels that include tens of millions of SNPs, we are developing an alternative method for querying reference data. This approach is expected to improve retrieval speed and scalability.

Additionally, we plan to implement more advanced PRS methods, such as PRS-CSx and LDpred, to give users more options to choose from. We will continuously update Genal as these optimizations and new functionalities are developed and integrated.

## 3 Example

In the Supplementary Material, we provide a tutorial along with an example in which we construct a PRS for systolic blood pressure and examine its genetic influence on the risk of all-cause stroke using MR. This analysis utilizes publicly available GWAS summary statistics. For the MR analyses, we achieve results that match those obtained with TwoSampleMR (Hemani *et al.* 2018), the most commonly used tool for such analyses. Additionally, we present a Python-based parallel implementation of MR-PRESSO. Our results align with those from the original R version of MR-PRESSO, and the method implemented in Genal is 87.5% faster. Specifically, for an analysis involving 1499 SNPs and 30 000 iterations of random data generation on an AMD Ryzen 5900× CPU with 12 cores at 3.7 GHz, the original R version required 15 h and 50 min, whereas our Genal version, completed the task in 1 h and 58 min.

## Supplementary Material

vbae207_Supplementary_Data

## Data Availability

The package is available on Pypi (https://pypi.org/project/genal-python/), the code is openly available on Github with a tutorial: https://github.com/CypRiv/genal, and the documentation can be found on readthedocs: https://genal.rtfd.io.
